# Diversity, Equity and Inclusivity‐ Low and Middle Income countries and the Lancet commission

**DOI:** 10.1002/alz.085064

**Published:** 2025-01-09

**Authors:** Gill Livingston, Jonathan D Huntley, Kathy Y Liu, Sergi Costafreda Gonzalez, Geir Selbaek, Suvarna Alladi, David Ames, Sube Banerjee, Alistair Burns, Carol Brayne, Nick C Fox, Cleusa P Ferri, Laura N. Gitlin, Robert J Howard, Helen C Kales, Mika Kivimäki, Eric B Larson, Noeline Nakasujja, Kenneth Rockwood, Quincy M Samus, Kokoro Shirai, Archana Singh‐Manoux, Lon S. S. Schneider, Sebastian Walsh, Yao Yao, Andrew Sommerlad, Naaheed Mukadam

**Affiliations:** ^1^ University College London, London United Kingdom; ^2^ University of Exeter, Exeter United Kingdom; ^3^ Division of Psychiatry, University College London, London United Kingdom; ^4^ University of Oslo, Oslo Norway; ^5^ Oslo University Hospital, Oslo Norway; ^6^ Norwegian National Advisory Unit on Aging and Health, Vestfold Hospital Trust, Tønsberg Norway; ^7^ National Institute of Mental Health and Neurosciences [NIMHANS], Bengaluru, Karnataka India; ^8^ AIBL Research Group, Perth and Melbourne Australia; ^9^ National Ageing Research Institute, Melbourne, VIC Australia; ^10^ University of Nottingham, Nottingham United Kingdom; ^11^ University of Manchester, Manchester United Kingdom; ^12^ Cambridge Public Health, University of Cambridge, Cambridge United Kingdom; ^13^ UK Dementia Research Institute at UCL, London United Kingdom; ^14^ Universidade Federal de São Paulo (UNIFESP), São Paulo, São Paulo/SP Brazil; ^15^ Drexel University, Philadelphia, PA USA; ^16^ Division of Psychiatry, University College London, London, London United Kingdom; ^17^ University of California, Davis, Sacramento, CA USA; ^18^ UCL Brain Sciences, University College London, London United Kingdom; ^19^ Department of Medicine, University of Washington, Seattle, WA USA; ^20^ College of Health Sciences, Makerere University, Kampala Uganda; ^21^ Nova Scotia Health, Halifax, NS Canada; ^22^ Johns Hopkins University, Baltimore, MD USA; ^23^ Osaka University, Osaka Japan; ^24^ Universite de Paris, INSERM, Paris France; ^25^ Alzheimer’s Disease Research Center, Keck School of Medicine, University of Southern California, Los Angeles, CA USA; ^26^ Peking University, Beijing China; ^27^ Camden and Islington NHS Foundation Trust, London United Kingdom

## Abstract

**Background:**

Data from high‐income countries (HICs) suggest a decline in age‐specific incidence rates of dementia. However, this has happened primarily in HICs, with low‐ and middle‐ income countries (LMICs) facing two main challenges: a higher burden of risk factors and, in general, a faster ageing population. Most people with dementia live in LMICs, and this is set to increase, thus requiring urgent and robust action to prevent, treat and support people with dementia and their families.

**Methods:**

We, in the Lancet Commission, reviewed the most recent literature, and conducted a new metanalysis on worldwide dementia risk. We have calculated worldwide figures, although there may be variation in different ethnic and socioeconomic groups.

**Results:**

Dementia studies are overwhelmingly from HICs, and there is a tendency to recruit people of European origin with higher education and socioeconomic status, with few people from ethnic minority groups. The same applies in respect of worldwide clinical trials, including multicomponent interventions to reduce risk, biomarker research and pharmacological/non‐pharmacological interventions.

Although most interventions are developed in HICs, culturally adapted interventions seem to be as effective in LMICs as in their original context, as long as the interventions’ core components are not compromised in the adaptation process.

**Conclusion:**

Policy interventions can improve dementia prevention, particularly in LMICs and in minority and lower level socio‐economic groups ‐ precisely the people who have the greatest burden of modifiable risk and are more likely to develop dementia.

Dementia prevention efforts should be tailored to the needs of the different countries and different groups within countries. Trials and research databases should aim for sociodemographic diversity to reflect real life populations.

Although evidence‐based interventions developed in HICs can be effective in LMICs, there is often a lack of healthcare infrastructure and resources to deliver them. Moreover, cultural differences can make them inappropriate or less effective. Interventions should be developed in partnership with local communities to ensure their appropriateness in respect of the culture, beliefs and practises which vary within and between countries.

